# Association between human cartilage glycoprotein 39 (YKL-40) and arterial stiffness in essential hypertension

**DOI:** 10.1186/1471-2261-12-35

**Published:** 2012-05-29

**Authors:** Wei-hong Ma, Xiu-ling Wang, Yi-meng Du, Yi-biao Wang, Yan Zhang, De-e Wei, Lin-lin Guo, Pei-Li Bu

**Affiliations:** 1 Department of Cardiology, The Second Hospital of Shandong University, Jinan, 250033, China; 2 Key Laboratory of Cardiovascular Remodeling and Function Research, Chinese Ministry of Education and Chinese Ministry of Public Health, Department of Cardiology, Qilu Hospital, Jinan, 250012, China; 3 Department of Cardiology, The Central Hostpital of Jinan City of Shandong University, Jinan, 250013, China

## Abstract

**Background:**

YKL-40, a proposed marker of inflammation and endothelial dysfunction, is associated with atherosclerosis and an increased cardiovascular mortality in the general population. However, the relationship between YKL-40 and arterial stiffness in hypertensive patients has not been adequately assessed.

**Methods:**

The relationship between serum levels of YKL-40 and arterial stiffness was evaluated in 93 essential hypertensive subjects and 80 normal subjects. Essential hypertensive subjects were divided into two groups based upon urinary albumin-to-creatinine ratio (ACR): nonmicroalbuminuric group, (ACR <30 mg/g, *n* = 50) and microalbuminuric group (ACR ≥30 mg/g, *n* = 43). Large artery wall stiffness was assessed by measuring femoral arterial stiffness and carotid-femoral pulse wave velocity (cf-PWV). Serum levels of YKL-40 were determined by enzyme-linked immunosorbent assay (ELISA).

**Results:**

The study demonstrated that YKL-40,cf-PWV and femoral arterial stiffness were increased significantly (*P*<0.05) in the hypertensive group compared with normal controls. These measurements were also increased significantly ( *P*<0.05) in the microalbuminuric group compared with the nonmicroalbuminuric group. YKL-40 was positively correlated with cf-PWV( *r* = 0.44, *P* = 0.000) and femoral arterial stiffness ( *r* = 0.42, *P* =0.001). Multiple linear stepwise regression analysis showed that YKL-40 was the impact factor of arterial stiffness ( *P*<0.05).

**Conclusion:**

YKL-40 levels are elevated in essential hypertension subjects with an independent association between increasing YKL-40 levels and increasing arterial stiffness. The study suggests it played a positive role of YKL-40 in the progressing vascular complications in patients with essential hypertension.

## Background

Hypertension is a common cardiovascular disease. Hypertension often initiates and accelerates the progression of macrovascular events by increasing arterial wall stiffness. YKL-40 has been suggested to be a potential biomarker of inflammation and endothelial dysfunction. Biomarkers of inflammation and endothelial dysfunction are associated with cardiovascular disease. Arterial stiffening may be a mechanism through which inflammation and (or) endothelial dysfunction lead to cardiovascular disease. Therefore, the measurement of arterial wall stiffness and serum levels of YKL-40 may be important strategies for the prevention and early treatment of cardiovascular events.

Although YKL-40 has been proposed to be a new marker of inflammation, atherosclerosis and endothelial dysfunction in neoplastic, cardiovascular and metabolic diseases, it has not been studied in the context of essential hypertension . The present study investigated the association between the serum levels YKL-40 and large artery compliance in subjects with essential hypertension compared with normal subjects.

## Methods

### Subjects

Ninety-three essential hypertensive untreated subjects in Qilu Hospital and The Second Hospital of Shandong University were enrolled in this study. The diagnosis was made in accordance with the 2007 Guidelines for the Management of Arterial Hypertension of the European Society of Hypertension (ESH) and the European Society of Cardiology (ESC) [1]. Blood pressures were measured by three separate verifications. Subjects with secondary hypertension, diabetes, chronic nephrosis, hyperlipemia, cardiovascular diseases and/or autoimmune diseases were excluded from the study.

The subjects with essential hypertension were divided into two groups, based upon the urinary albumin-to-creatinine ratio (ACR): nonmicroalbuminuric (ACR < 30 mg/g) and microalbuminuric (ACR ≥ 30 mg/g). Normotensive subjects served as controls.

The nonmicroalbuminuric (NMA) group included 50 patients (26 females and 24 males), with a mean age of 59.2 ± 12.1 years (range 32 – 79 years). The mean systolic blood pressure (SBP) was 157.0 ± 16.9 mmHg(range 115-200 mmHg) and the mean diastolic blood pressure (DBP) was 95.9 ± 10.8 mmHg(range 69-113 mmHg). The microalbuminuric (MA) group included 43 subjects (24 female, 19 male), with a mean age of 57.9 ± 13.6 years (range 30 – 78 years). SBP was 169.7 ± 24.3 mmHg (range 130-240 mmHg) and DBP was 103.2 ± 20.5 mmHg (range 70-146 mmHg).

There were 80 normotensive controls (39 females, 41 males), with a mean age of 57.7 ± 13.4 years (range 24 – 84 years). SBP was 115.9 ± 10.67 mmHg (range 90-135 mmHg) and DBP was 76.8 ± 5.9 mmHg (range 65-90 mmHg).

Femoral arterial systolic diameter (Ds), diastolic diameter (Dd), stiffness, tensity and distensibility (calculated), pulse wave velocity (PWV) and serum levels of YKL-40 were measured for all subjects. The clinical information for all the subjects was collected include, fasting blood glucose(FBG), total triglyceride(TG), total cholesterol (TC), high density lipoprotein cholesterol (HDL), low density lipoprotein cholesterol (LDL), blood urea nitrogen (BUN), serum creatinine (Cr), heart rate, height, weight, etc. (Table1 and Table2) This belongs in the results: The clinical information of the three groups have no difference, so the three groups have comparability. This study was approved by ethics committee of the Second Hospital of Shandong University. All subjects provided written consent.

**Table 1:** **Basic characteristics**$\left(\bar{X}\pm s\right)$

	**essential hypertensive groups**		
	**nonmicroalbuminuric**	**microalbuminuric**	**healthy controls**
	**(*n* = 50)**	**(*n* = 43)**	**(*n* = 80)**
Age	59.21 ±12.09	57.86 ± 13.63	57.69 ± 13.38
Sex distribution (M/F)	24/26	19/24	41/39
MA(mg/g)	19.92 ± 4.44^*^	81.44 ± 26.77^*△^	16.97 ± 5.84
Cr(mg/dl)	0.85 ± 0.18^*^	0.93 ± 0.24^*^	0.74 ± 1.67
GFR(ml.min^-1^.1.73 m^-2^)	89.54 ± 22.48^*^	88.73 ± 27.90^*^	113.85 ± 36.76
FPG(c/mmol.L^-1^)	5.79 ± 1.42	5.82 ± 1.23	5.38 ± 0.74
TC(c/mmol.L^-1^)	5.30 ± 1.05^*^	5.23 ± 0.98^*^	4.59 ± 0.67
HDL (c/mmol.L^-1^)	1.42 ± 0.38	1.33 ± 0.28^*^	1.50 ± 0.36
LDL(c/mmol.L^-1^)	2.94 ± 0.92^*^	3.03 ± 0.89^*^	2.25 ± 0.54
TG (c/mmol.L^-1^)	1.80 ± 1.29	2.02 ± 1.34	1.55 ± 0.72
BUN (c/mmol.L^-1^)	5.46 ± 1.22	5.67 ± 2.08	4.81 ± 0.86
BMI(kg/m^2^)	26.16 ± 2.72^*^	27.92 ± 3.04^*△^	23.68 ± 3.21
SBP(p/mmHg)	157.03 ± 16.90^*^	169.74 ± 24.28^*△^	115.94 ± 10.66
DBP(p/mmHg)	95.87 ± 10.76^*^	103.18 ± 20.53^*△^	76.81 ± 5.93
PP(p/mmHg)	59.75 ± 17.18^*^	66.13 ± 20.24^*^	39.13 ± 7.81

Data are presented as mean ± SD or number. *MA*: microalbuminuria; *Cr* : serumcreatinine; *GFR* : the glomerular filtration rate; *FPG* : fasting blood glucose; *TC* : total cholesterol; *HDL* : high density lipoprotein cholesterol; *LDL* : low density lipoprotein cholesterol; *T*G : triglyceride; *BUN* : blood urea nitrogen; *BMI*: body mass index; *SBP*: systolic pressure; *DBP*: diastolic pressure; *PP*:pulse pressure;^*^P<0.05 vs healthy controls, ^△^*P*<0.05 vs nonmicroalbuminuric.

**Table 2:** **Changes in indexes of ultrasound of femoral artery, PWV and YKL-40 among different groups**$\left(\bar{X}\pm s\right)$

	**essential hypertensive groups**		
	**nonmicroalbuminuric**	**microalbuminuric**	**healthy controls**
	**(n = 50)**	**(n = 43)**	**(n = 80)**
IMT(mm)	0.87 ± 0.14^*^	1.08 ± 0.16^*#^	0.65 ± 0.14
PSV(cm/s)	96.25 ± 26.96	95.97 ± 25.28	95.34 ± 25.58
EDV(cm/s)	15.17 ± 5.12	15.46 ± 5.89	17.13 ± 6.32
stiffness	455.75 ± 199.38^*^	614.59 ± 234.38^*#^	382.14 ± 165.23
tensity	14.93 ± 7.42	12.01 ± 6.05^*#^	15.38 ± 7.58
distensibility	0.69 ± 0.21	0.46 ± 0.17^*#^	0.96 ± 0.31
cf-PWV(ms)	10.40 ±1.98^*^	12.26 ± 2.13^*#^	8.67 ± 2.90
YKL-40(ng/ml)	61.63 ± 18.58^*^	98.78 ± 19.83^*#^	37.85 ± 14.12

Data are presented as mean ± SD. *IMT*: intima-media thickness; *PSV*: peak systolic velocity; *EDV*: end diastolic velocity; *PWV*: pulse wave velocity; ^*^P<0.05 vs healthy controls; ^#^*P*<0.05 vs nonmicroalbuminuric.

### Ultrasound measurement

The Doppler sample volume was placed 1.5 cm superior to the forked point of femoral artery. The femoral arterial intima-media thickness (IMT), systolic diameter (Ds, at peak of T wave on electrocardiogram), diastolic diameter (Dd, at peak of R wave on electrocardiogram), the peak systolic velocity (PSV), end diastolic velocity (EDV) were tested at 3 different points. Femoral arterial stiffness, tensity and distensibility were calculated. Stiffness = Pulse pressure(PP) × Dd/(Ds-Dd), tensity = (Ds-Dd) × 100/Dd, distensibility = (Ds^2^-Dd^2^) × 100/(Dd^2^ × PP)[2,3]. One experienced reader who was unaware of the case control status of the subjects performed all measurements, using a diagnostic ultrasound system (iE33, PHILIPS Ultrasound,Washington, US) with a L11-3 probe of 3-11 MHz.

### Clinical data

Blood pressures were obtained from the right arm of each subject using the floor stand model mercurial sphygmomanometer (YUTU, Shanghai, China) in a sedate environment after resting for 15 min. Blood pressures were repeated twice, at two minute intervals, and the mean value calculated. Mean pulse pressure(PPv = SBP – DBP) was calculated for each subject. Anthropometric measurements were obtained, and body mass index(BMI)was calculated by the following function: BMI = weight(kg)/height^2^(m^2^). Venous blood samples were obtained in the morning after a 12 h fast. Tests were performed by Autonomic Biochemical Analyzer (DVI1650, BAYER, GERMANY), including FBG, TG,TC, HDL, LDL, BUN and sCr,. The glomerular filtration rate(GFR)was calculated: GFR(ml.min-1.1.73 m-2) = 186 × (Cr)-1.154 × (age)-0.203 × (0.742 female).

### ACR determination

An untimed urine sample was collected during visit 4. Aliquots were frozen within 12 h and stored at -70°C. Albumin and creatinine concentrations were measured in the biochemical laboratory of Qilu Hospital, the microalbuminuria was tested by immunoturbidimetry (assay sensitivity, 2.0 mg/l) and the urinary creatinine by modified Benedict-Behre. The ACR was calculated automatically. ACR (mg/g) was categorized into NMA group (ACR<30 mg/g, *n* = 50) and MA group (ACR ≥30 mg/g, *n* = 43) [4].

### Serum YKL-40 determination

The serum level of YKL-40 was determined by enzyme-linked immunosorbent assay (ELISA, B.G,Blue Gene, assay sensitivity, 0.01 ng/ml). . Blood samples were centrifuged at 3000 rpm for 5 min after being left under room temperature for 30 min. The supernatant was removed, placed in Eppendorf tuber respectively and stored at -80°C conditions for further test. Each reagent and blood sampled was brought to room temperature for analysis.

### Cf-PWV measurement

cf-PWV measurements were assessed by an automatic device (Complier SP, Bartech Medical, France) with the subjects supine after resting for 15 min. The 2 press receptors were placed at the positions of bilateral carotid artery and femoral artery with most obvious pulse waves respectively, when the waves were stable bilateral cf-PWV were recorded.

### Statistical analysis

Statistical analyses were performed using SPSS 17.0 statistical software package The normally distributed quantitative variables were expressed as mean ± standard deviation . Comparisons among the 3 groups were done by ANOVA. The association of each variable with femoral arterial stiffness was assessed by Pearson correlation coefficients, and trend analysis was performed by Spearman rank correlation. Application of multiple stepwise regression analysis the affecting factors of femoral arterial stiffness, distensibility and PWV, *P*<0.05 for the difference was statistically significant.

## Results

### Comparison of common clinical data

There was no significant difference between the hypertensive subjects and normal controls for the following variables: age, gender, fasting blood glucose (FBG), triglycerides (TG), and blood urea nitrogen (BUN). The hypertensive patients were significantly more likely to have an elevated creatinine (Cr), total cholesterol (TC), low density lipoprotein cholesterol (LDL), body mass index(BMI), systolic pressure (SBP), diastolic pressure (DBP) and pulse pressure (PP) (*P*<0.05),but glomerular filtration rate (GFR) was decreased in comparison to normal controls. BMI, SBP and DBP were significantly higher for subjects with MA compared to NMA subjects. (Table1)

### The comparison of indicators arterial function

The IMT, stiffness of femoral artery, and cf-PWV were markedly higher in hypertensive groups than in controls which is the same in the comparison between MA group and NMA group.(*P*<0.01, *P*<0.05) While tensity and distensibility of femoral artery were significantly lower in hypertensive groups than in controls ( *P*<0.01). PSV and EDV did not show significant difference among groups ( *P >* 0.05) (Table2).

### The comparison of serum YKL-40

**Figure 1:**
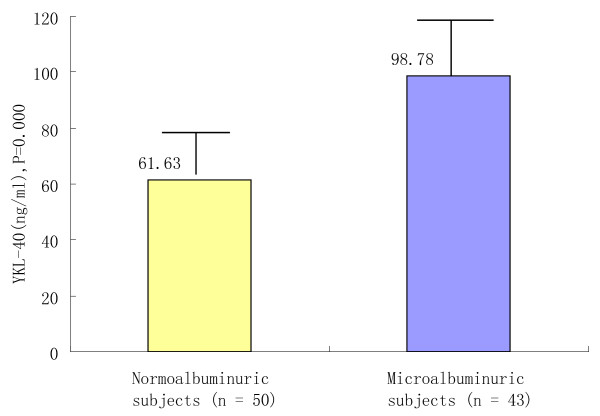
**Serum level of YKL-40 in nonmicroalbuminuric and microalbuminuric essential hypertensives.** Serum level of YKL-40 was compared in nonmicroalbuminuric and microalbuminuric essential hypertensives. The level of YKL-40 in nonmicroalbuminuric patients is 61.63 ± 18.58, while the level of YKL-40 in microalbuminuric group is 98.78 ± 19.83. YKL-40 was increased in microalbuminuric patients than in nonmicroalbuminuric subjects ( *P*<0.05).

The serum YKL-40 results of each group: NMA group (61.63 ± 18.58 ng/ml), MA group (98.78 ± 19.83 ng/ml) and healthy controls (37.85 ± 14.12 ng/ml). YKL-40 was significantly higher in hypertensive group than in control group (*P*<0.05) and further increased in microalbuminuric patients than in nonmicroalbuminuric subjects ( *P*<0.05) (Table2, Figure1).

### Correlation analysis between YKL-40 and the correlated indexes

**Figure 2:**
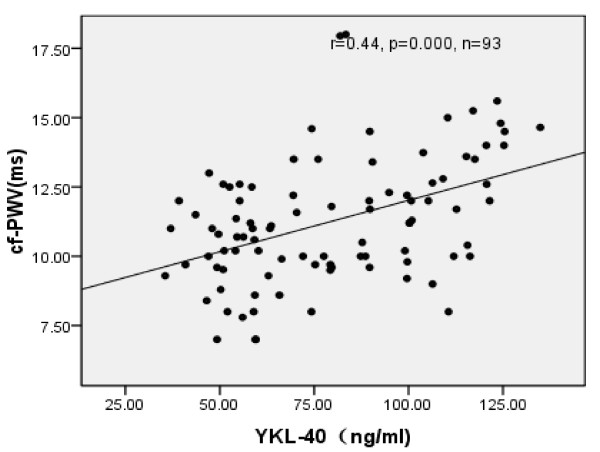
**Scatter diagram of YKL-40 and cf-PWV in normoalbuminuric and microalbuminuric hypertensions.** Spearman correlation of circulating plasma levels YKL-40 and cf-PWV. The level of YKL positively correlate with **cf-PWV** in normoalbuminuric and microalbuminuric hypertensions ( *r =* 0.44, *P* = 0.00).

**Figure 3:**
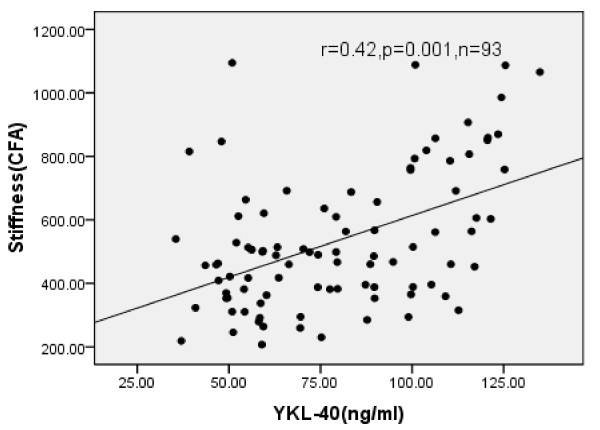
**Scatter diagram of YKL-40 and stiffness(CFA) in normoalbuminuric and microalbuminuric hypertensions.** Spearman correlation of circulating plasma levels YKL-40 and stiffness. The level of YKL positively correlate with stiffness in normoalbuminuric and microalbuminuric hypertensions ( *r* = 0.42, *P* = 0.001).

Significant positive correlations were noted between YKL-40 and the stiffness of the femoral artery (*r* = 0.42, *P*<0.01), cf-PWV ( *r* = 0.44, *P*<0.01), MA,IMT, BMI and age( *P*<0.05), while tensity and distensibility were significantly negatively correlated to stiffness in hypertensive group ( *P*<0.05) (Table3, Figure2, Figure3).

**Table 3:** **correlation analysis between YKL-40 and the correlated indexes (*R***  **= 0.634) (*n***  **= 93)**

**Items**	**Correlation coefficient(r)**	***P*-value**
Stiffness	0.42	0.001
PWV	0.44	0.000
MA	0.51	0.000
Tensity	-0.22	0.034
Distensibility	-0.38	0.001
IMT	0.32	0.002
BMI	0.21	0.040
Age	0.11	0.300

Correlation analysis between stiffness and the correlated indexes. *IMT*: intima-media thickness; *PWV*: pulse wave velocity; *MA*: microalbuminuria; *BMI*: body mass index.

### YKL-40 was the impact factor of arterial stiffness

With the stiffness and distensibility of the femoral artery and pulse wave velocity (PWV) as dependent variables, age, blood pressure(SBP, DBP), BMI, blood lipid (TG, HDL, LDL), fasting blood glucose and YKL-40 as independent variables by using the multivariate linear stepwise regression analysis. The YKL-40 on femoral artery stiffness contribution rate was 36.9% (*F* = 14.781, *P* =0.001, Table4). On distensibility of contribution ratio was 36.9% (*F* = 10.743, *P* =0.000, Table5),on the PWV was 43.8% (*F* =21.580, *P* =0.000,Table6). 翻译结果重试http://

**Table 4:** **Multivariate linear stepwise regression of femoral arterial stiffness and the correlated indexes (*R***^**2**^ **= 0.402)(*n***  **= 93)**

**Items**	**partial regression**	**standardized partial**	***t* - value**	***P*- value**
	**coefficientβ**	**regression coefficientβ**		
YKL-40	0.006	0.37	4.28	0.001
Age	0.011	0.36	4.26	0.000
BMI	0.031	0.23	2.72	0.008
TG	-0.059	-0.17	-2.05	0.043
SBP	-0.001	-0.064	-0.564	0.574
DBP	0.000	-0.022	-0.212	0.833
HDL	0.000	-0.005	-0.053	0.958
LDL	0.034	0.076	0.868	0.388
FPG	-0.018	-0.046	-0.490	0.625

Multivariate linear stepwise regression of femoral arterial stiffness and the correlated indexes (*R*^*2*^ = 0.402)(*n* = 93). BMI: body mass index; *TG* : triglyceride; *SBP*:systolic pressure; *DBP*: diastolic pressure; *HDL*: high density lipoprotein cholesterol; *LDL* : low density lipoprotein cholesterol; *FPG* : fasting blood glucose.

**Table 5:** **Multivariate linear stepwise regression of the femoral arterial distensibility and the correlated indexes (*R***^**2**^ **= 0.193)(*n***  **= 93)**

**Items**	**partial regression**	**standardized partial**	***t*- value**	***P*- value**
	**coefficient *β***	**regression coefficient*β***		
YKL-40	0.001	-0.385	-3.635	0.000
Age	0.002	-0.003	-0.025	0.980
BMI	0.009	0.050	0.480	0.633
TG	0.023	0.020	0.206	0.837
SBP	0.002	-0.126	-0.981	0.329
DBP	0.002	0.231	1.916	0.059
HDL	0.002	0.054	0.516	0.607
LDL	0.030	-0.015	-0.154	0.878
FPG	0.027	-0.225	-0.211	0.037

Multivariate linear stepwise regression of femoral arterial distensibility and the correlated indexes (*R*^*2*^ = 0.193)(*n* = 93).

**Table 6:** **Multivariate linear stepwise regression of the pulse wave velocity(logPWV) and the correlated indexes (*R***^**2**^ **= 0.192)(*n***  **= 93)**

**Items**	**partial regression**	**standardized partial**	***t*- value**	***P*- value**
	**coefficient*β***	**regression coefficient*β***		
YKL-40	0.009	0.401	3.735	0.000
Age	0.018	0.012	0.116	0.908
BMI	0.080	0.086	0.802	0.425
TG	0.195	0.026	0.265	0.792
SBP	0.015	0.104	0.798	0.427
DBP	0.017	-0.062	-0.509	0.612
HDL	0.014	0.054	0.514	0.609
LDL	0.259	0.038	0.378	0.706
FPG	0.234	-0.006	-0.053	0.957

Multivariate linear stepwise regression of the pulse wave velocity (PWV) and the correlated indexes (*R*^*2*^ = 0.192)(*n* = 93).

抱歉, 系统响应超时, 请稍后再试

· 支持中英, 中日在线互译

· 支持网页翻译, 在输入框输入网页地址即可

· 提供一键清空, 复制功能, 支持双语对照查看, 使您体验更加流畅

Analysis showed that YKL-40 was the impact factor arterial stiffness (*P*<0.05).

## Discussion

Hypertension,a progressing in chronic inflammation and cardiovascular syndrome with various causes, results in functional and structural changes of heart and arterial vessels. The evaluation of vascular damage caused by hypertension was made in two parts: functional and structural test, while the vascular functional abnormality was mainly characterized as degeneration of arterial elasticity. Femoral arterial stiffness, tensity and distensibility and cf-PWV could help to observe structural changes of vascular wall directly and evaluate vascular elasticity objectively.

YKL-40 is a 40 kDa heparin- and chitin-binding glycoprotein which is secreted.

Invitro by a variety of cells. InvivoYKL-40 is found in subpopulations of macrphages and VSMCs in different tissues with inflammation and extracellular matrix remodeling as in atherosclerotic plaques [5]. YKL-40 has been suggested to be a potential biomarker of inflammation and endothelial dysfunction [5]. It is a useful screening tool because it is detected in early stage subclinical disease, and it also appears to have the potential of becoming a significant prognosticator of cardiovascular events and mortality [6] .

Our results demonstrate YKL-40 was increased significantly in essential hypertension group and further increased in the MA subjects compared with NMA subjects. MA is a marker of target organ damage (TOD) in hypertensive patients [7]. Decreased eGFR is associated with an increased risk of arterial stiffness in community residents [8]. Positive correlations were noted between YKL-40 and MA, IMT. The common femoral artery (CFA) IMT was demonstrated to be the most sensitive descriptor [9]. It implied YKL-40 might be used as a viewing window to observe subclinical target organ damage of hypertension. Serum level of YKL-40 was significantly associated with femoral stiffness, tensity and distensibility. YKL-40 was an independent predicator of functional changes of artery, implicating that high level of YKL-40 affects arterial compliance. To the best of our knowledge, we detected there is a strong relationship between increased serum level of YKL-40 and essential hypertension for the first time. Hypertension is intimate correlated with inflammation. YKL-40 is a marker of inflammation and endothelial dysfunction [10].

Femoral arterial stiffness could access arterial elasticity directly. Many factors take part in elevation of arterial stiffness. The participation of YKL-40 in inflammatory states and vascular processes implies that YKL-40 may play a role in endothelial dysfunction and atherosclerosis. YKL-40 is an inflammatory glycoprotein involved in endothelial dysfunction by promoting chemotaxis, cell attachment and migration, reorganization and tissue remodelling as a response to endothelial damage [11]. We found microalbuminuric patients’ YKL-40 and stiffness increased and tensity and distensibility decreased were comparable with nonmicroalbuminuric patients. YKL-40 was positively correlated with femoral arterial stiffness and it was the impact factor of stiffness of femoral artery. Subendothelial abnormal deposit and deformation of lipid resulted from inflammatory response and oxidative stress,resulted in endothelial proliferation,followed by fibrillation and elevation in endothelial thickness. All-layer-involved diffuse and continuous increase in arterial stiffness, vascular dilation and elevation in endothelial thickness provided basis for formation of artherosclerosis. cf-PWV is a common method to evaluate the function of vessels and a classic index to evaluate the stiffness of artery [12]. Mori J Krantz et al. [13] reported that PWV was associated with preclinical carotid atherosclerosis independent of Framingham risk factors in cross-sectional study of a mixed-ethnicity population. PWV is associated with preclinical atherosclerosis among a Latino-predominant population. PWV as an indicator of arterial distensibility, may play an important role in the stratification of patients based on the cardiovascular risk. PWV inversely correlates with arterial distensibility and relative arterial compliance. In our study, microalbuminuric group had higher levels of cf-PWV compared with those of nonmicroalbuminuric group. The MA subjects also had significantly lower arterial tensity and distensibility compared with controls. YKL-40 was positively correlated with cf-PWV, demonstrating a relationship probably existed betweenYKL-40 and cf-PWV. Multiple linear regression analysis confirmed YKL-40 was femoral artery stiffness and cf-PWV independent factors. It can be used a valuable index to evaluated arterial function and further predict complication in essential hypertension.

YKL-40, MA, cf-PWV and the femoral arterial stiffness increase progressively with an increase in SBP and DBP. Recent work has identified YKL-40 as a promoter of angiogenesis in neoplasms, including activating the mitogen activated protein kinase/extracellular signal regulated kinase (MAPK/ERK) pathway in endothelial cells [14]. Activated MAPK and/or Akt may be negatively regulated by EGFR. Both pathways are required for the cells to complete mitosis and the activation of these pathways stimulates the growth of connective tissue cells [15]. Next we will investigate the interaction between MAPK signaling and YKL-40 in essential hypertension.

## Conclusions

In summary, the present investigation demonstrated that a positive correlation exists between YKL-40 and femoral arterial stiffness and cf-PWV. YKL-40 was the impact factor of arterial wall stiffness, the mechanism possibly related to arterial endothelium dysfunction. YKL-40 is sensitive indicators that could predict early target organ damage and evaluate arterial function in hypertension.

## Competing interests

The authors declare that they have no competing interests.

## Authors’ contributions

WH Ma was the lead on ultrasound measurement and carried out primary drafting of the paper, study design and all of the statistical analyses. PLB contributed to study design and review manuscript. XLW contributed to literature searches and the investigation of the patients. YMD was involved in quality control. YBW was the senior statistician and provided critical content review. YZ was responsible for translation. DEW and LLG contributed to blood sample collection and part of data analyses. All authors read and approved the final manuscript.

## Pre-publication history

The pre-publication history for this paper can be accessed here:

http://www.biomedcentral.com/1471-2261/12/35/prepub
